# The Quality of the Parent–Child Relationship in the Context of Autism: The Role of Parental Resolution of the Child’s Diagnosis, Parenting Stress, and Caregiving Burden

**DOI:** 10.3390/ejihpe15070142

**Published:** 2025-07-18

**Authors:** Annalisa Levante, Chiara Martis, Flavia Lecciso

**Affiliations:** 1 Department of Human and Social Sciences, University of Salento, 73100 Lecce, Italy; chiara.martis@unisalento.it (C.M.); flavia.lecciso@unisalento.it (F.L.); 2 Laboratory of Applied Psychology, Department of Human and Social Sciences, University of Salento, 73100 Lecce, Italy

**Keywords:** autism, parenting stress, caregiving burden, resolution of the diagnosis, parent-child relationship

## Abstract

**Background.** Parents of autistic children face challenges that can negatively affect the quality of the parent–child relationship. This study aimed to explore the potential protective role of parental resolution about positive (closeness) and negative (conflict and dependence) aspects of the parent–child relationship, with parenting stress and caregiving burden as mediators. **Methods**. A cross-sectional study (ethical approval: CE n. 92949) was conducted with 51 Italian parents of autistic children. A multiple mediation model was tested. **Results**. Parental resolution had a significant total effect (β = 0.012; BootLLCI = 0.002; BootULCI = 0.024) and a significant direct effect on the parent–child relationship (β = 0.223; BootLLCI = 0.058; BootULCI = 0.389), indicating that resolving the child’s diagnosis could potentially influence parents’ perceptions of their relationship with their child, possibly leading to views of it being somewhat closer, experiencing fewer conflicts, and involving a lower degree of dependence. An indirect effect via parenting stress was also significant (β = −0.130; BootLLCI = −0.009; BootULCI = −0.291), while caregiving burden did not show a mediating effect. **Conclusion**: Despite the exploratory and cross-sectional nature of this study, the findings highlight the importance of promoting family well-being in the context of autism. The findings may inform future research on parental resources and guide clinicians in developing intervention programmes to mitigate the emotional impact of receiving a child’s autism diagnosis.

## 1. The Quality of the Parent–Child Relationship in the Context of Autism

A large number of studies (Repetti et al., 2002; Tamis-LeMonda et al., 2004; Fonagy et al., 2016; Solak Arabaci & Demircioğlu, 2021; Pianta & Steinberg, 1992; Pianta & Stuhlman, 2004; Marchetti et al., 2013; Lecciso et al., 2011) have shown that the quality of the parent–child relationship significantly influences several aspects of a child’s development. These aspects range from language acquisition and cognitive abilities to Theory of Mind skills and socio-emotional growth, with even academic success being affected. The theoretical framework underpinning the discussion of the parent–child relationship is Bowlby’s (1969) Attachment Theory. According to Bowlby, a child’s early experiences of parental responsiveness involve seeking physical proximity and exploring the environment, which, in turn, shape the child’s expectations of receiving help and support from the caregiver. Throughout development, the child internalises these parental behaviours and forms internal working models—mental representations of attachment bonds that guide the individual in establishing new affective relationships with other significant figures across their lifespans, such as educators, friends, teachers, and romantic partners (Bowlby, 1969; Cassibba et al., 2017; Verhage et al., 2016). Based on this theoretical foundation, parental characteristics play an essential role in shaping and maintaining the parent–child relationship.

Critical developmental contexts, especially those involving a child with a disability, may give rise to stressful challenges that compromise the parent–child relationship (Coppola et al., 2012; Hickey et al., 2020; Totsika et al., 2020). Autistic traits in particular are often associated with increased caregiving demands, which may negatively affect the quality of the parent–child relationship (Davis & Carter, 2008; Da Paz & Wallander, 2017; Hirschler-Guttenberg et al., 2015). Autism is a pervasive and lifelong neurodivergent condition characterised by an array of abilities accompanied by some challenges in social communication and repetitive behaviours (American Psychiatric Association, 2013). A positive parent–child relationship may serve as a cornerstone for an autistic child’s social, emotional, and cognitive development. For instance, a warm parent–child relationship may result in the child’s behavioural adjustment and improved competence (Koren-Karie et al., 2009). Additionally, emotional support and cohesion within the parent–child relationship positively influence the development and improvement of children’s social skills (Haven et al., 2014). Parents of autistic children who report a close parent–child relationship often adapt their communication styles, establish consistent routines, and use positive reinforcement strategies to nurture their child (Johnson & Lee, 2021). Similarly, parental involvement is positively associated with more successful therapy outcomes (Solomon et al., 2008; E. Smith et al., 2022; Colombi et al., 2023).

Evidence of the positive cascade effects related to a positive parent–child relationship highlights the need to investigate the factors affecting such a relationship in the context of autism. Although parental factors influencing the quality of the parent–child relationship have already been examined in autism research, studies involving this population remain relatively limited.

The following sections will summarise the existing literature on the role of parental resolution of a child’s diagnosis as a potential personal resource that may positively affect the quality of the parent–child relationship. Parenting stress and caregiving burden will be explored as contributing factors.

### 1.1. Parental Factors Affecting the Quality of the Parent–Child Relationship in the Context of Autism

#### 1.1.1. Parental Resolution of a Child’s Autism Diagnosis and the Quality of the Parent–Child Relationship

When parents receive their child’s diagnosis, the expectations they may have held for a typically developing and healthy child need to shift to align with the reality of parenting a child with a disability (Luong et al., 2009). Parents have to deal with the new situation, which concerns not only their child’s condition, but also their experience of parenthood (Kamphorst et al., 2018). Letting go of the expectations associated with typical development causes emotional pain and may be compared to a grieving process (Main & Hesse, 1990). The parental reaction to the diagnosis is often characterised by initial shock and denial, followed by negative emotions such as guilt and shame (Kübler-Ross, 1973). This nonlinear process may lead either to resolution or a lack of resolution (Marvin & Pianta, 1996). The benefits of achieving resolution of the child’s diagnosis have been extensively demonstrated. Parents who resolve the diagnosis tend to reorganise their daily life and family routines, becoming aware of the child’s functioning (Oppenheim et al., 2009; Lecciso et al., 2013). By contrast, unresolved parents may experience persistent denial or anger, which can negatively affect family dynamics (Oppenheim et al., 2009; Lecciso et al., 2013). A lack of resolution may result in parents feeling overwhelmed, with them living in a state of permanent grieving (Marvin & Pianta, 1996).

In the context of autism, some studies have focused on the impact of parental resolution of the child’s diagnosis. Evidence has shown that parental resolution enhances parents’ coping strategies for dealing with stressors (Da Paz et al., 2018), increasing their supportive engagement during play interactions with their child through improved verbal and nonverbal scaffolding skills (Wachtel & Carter, 2008; Lecciso et al., 2013). A positive association between parental resolution and the quality of the parent–child relationship has also been demonstrated (Wachtel & Carter, 2008; Oppenheim et al., 2009; Lecciso et al., 2013). Parents who had resolved their child’s autism diagnosis reported a warm, emotionally close relationship and a secure attachment bond. Sher-Censor et al. (2017) also found that parents who had achieved resolution were more emotionally available towards their autistic children than unresolved parents. Due to the prevalence of unresolved status among parents of autistic children and its detrimental effects on the quality of the parent–child relationship (Sher-Censor & Shahar-Lahav, 2022), research targeting this issue represents a crucial public health concern.

#### 1.1.2. Parenting Stress and the Quality of the Parent–Child Relationship

Parenting stress refers to the psychological strain resulting from the demands associated with caring for a child (Abidin, 1992). Studies have highlighted that the main parental concerns relate to children’s behaviours and development (Akister & Johnson, 2004; Reijneveld et al., 2008). Unsurprisingly, parenting stress may increase dramatically when a child is diagnosed with a disability (Barroso et al., 2018). Parenting stress has been one of the most explored factors in research on families of autistic children. In this context, parents—mostly mothers (Tehee et al., 2009)—report higher levels of stress compared to parents of typically developing children or children with other developmental delays or disorders (Catalano et al., 2018; Estes et al., 2014; Bitsika et al., 2013; Hayes & Watson, 2013; Pisula, 2007; Seymour et al., 2013; Tomanik et al., 2004). The factors influencing the level of parenting stress may be related to both the children’s autistic traits and co-occurring behavioural problems (Maskey et al., 2013; Valicenti-McDermott et al., 2015; Hastings, 2003), as well as communication challenges, social isolation, and difficulties in self-care (Schieve et al., 2007). Other contributing factors to parenting stress include some sociodemographic characteristics, such as the parents’ gender. For instance, mothers generally report higher stress levels than fathers (e.g., Hastings, 2003). Although the determinants of parenting stress have been widely explored (e.g., Rivard et al., 2014), its impact on parental and child domains requires further investigation. Research involving families of autistic children suggests that parenting stress negatively affects parental problem-solving and coping strategies (Fingerman et al., 2020), with detrimental effects on treatment outcomes (Osborne et al., 2008). Little is known about the impact of parenting stress on the quality of the parent–child relationship. Davis and Carter (2008) highlighted that stressed parents reported dysfunctional and difficult interactions with their children, due to the children’s autistic traits. Similarly, Hickey et al. (2020) found that stressed parents reported low warmth and high conflict in the parent–child relationship. Although these findings offer valuable insights, further research on this issue is needed. No studies have yet explored the mediating role of parenting stress on the path between parental resolution of the child’s diagnosis and the quality of the parent–child relationship.

#### 1.1.3. Caregiving Burden and the Quality of the Parent–Child Relationship

Caregiving burden refers to the negative impact that providing care may have across various life domains when supporting vulnerable individuals (Given et al., 2001). Parents of children with disabilities report a greater caregiving burden compared to parents of typically developing children (Minhas et al., 2015). Caregiving for autistic children can be overwhelming and may have a significant impact on families (Renty & Roeyers, 2006), with families of autistic children experiencing poorer mental health than the general population (Ghanizadeh et al., 2009). Parents—especially mothers—of autistic children (Bello-Mojeed et al., 2013; L. E. Smith et al., 2010) spend more time providing care and less time engaging in leisure activities than parents of children without disabilities. Factors contributing to caregiving burden include parenting stress (Giallo et al., 2013; Almansour et al., 2013), the severity of the child’s autism (Patel et al., 2022; Stuart & McGrew, 2009; Dardas & Ahmad, 2014; Baykal et al., 2019), and several sociodemographic characteristics, such as the parents’ gender, low educational attainment, co-residence with the care recipient, and an increased number of caregiving hours (Adelman et al., 2014). The effects of high caregiving burden have been shown to be lower parental quality of life (Marsack-Topolewski & Church, 2019) and reduced life satisfaction (Cetinbakis et al., 2020). A study on parents of typically developing children reported a positive association between caregiving burden and parent–child conflict, and a negative association with parent–child closeness (Russell et al., 2020). Little is known about the direct and mediating impact of caregiving burden on the quality of the parent–child relationship in the context of autism.

**This Study**

The theoretical background outlined in the previous sections highlights the paucity of studies exploring the potential predictive role of parental resolution in the quality of the parent–child relationship in the context of autism, with parenting stress and caregiving burden as potential mediators.

**Figure 1:**
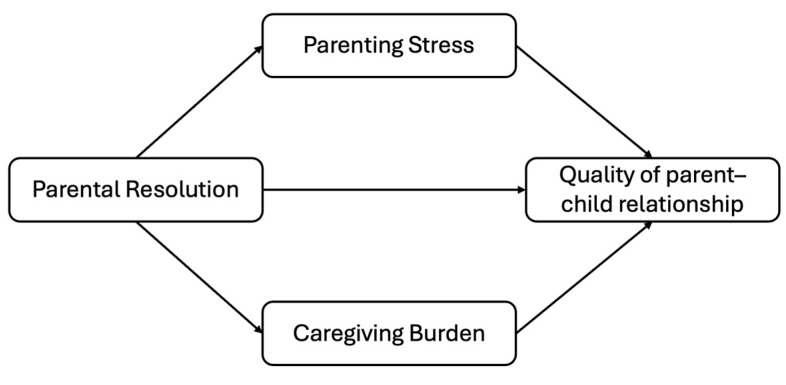
Hypothesised multiple mediation model.

Figure 1 shows the multiple mediation model developed to address the following research questions:

**RQ1**: Is parental resolution a potential predictor of a parent–child relationship characterised by closeness, low conflict, and reduced dependence through low levels of parenting stress?

**RQ2**: Is parental resolution a potential predictor of a parent–child relationship characterised by closeness, low conflict, and reduced dependence through low levels of caregiving burden?

## 2. Materials and Methods

### 2.1. Procedure and Statistical Plan

This study was part of a larger research project investigating parental and sibling attitudes in families with a member diagnosed with a disability (Levante et al., 2023; Levante et al., 2024; Lecciso et al., 2025a, 2025b). It was approved by the Ethics Committee for Research in Psychology (CE n. 92949) of the Department of Human and Social Sciences at the University of Salento, Italy. Prior to completing the online questionnaire designed for this study, each participant provided informed electronic consent. If consent was denied, the system automatically blocked access to the questionnaire. A snowball sampling strategy was used for data collection through major social media platforms, including WhatsApp, Instagram, and Facebook. The inclusion criteria for the parent sample were as follows: (1) being a parent aged 18 years or older; (2) having a child with a diagnosis of autism spectrum disorder; (3) being fluent in the Italian language; (4) having no disability.

Data were analysed using SPSS v. 25 (IBM Corp, 2023) and Jamovi (The Jamovi Project, 2024). The significance level was set at *p* < 0.050. No imputation techniques were applied for missing data, as all the questionnaire items were mandatory. The Gaussianity of the data distribution and the heterogeneity of variance were also tested.

Preliminary and descriptive analyses included a table reporting descriptive statistics for the study variables (mean, standard deviation, and theoretical range). Group comparisons were conducted to test differences in the study variables between parents of boys and girls. Correlational analyses were performed to examine associations between sociodemographic characteristics (e.g., child’s age, parents’ age) and the study variables. These analyses informed the selection of covariates for the subsequent analyses. The main analysis involved testing the study hypotheses (Figure 1) using Model 6 of the PROCESS macro. The multiple mediation model developed examined parental resolution of the child’s diagnosis as a potential predictor of the quality of the parent–child relationship, conceptualised as an aggregate variable comprising both positive (closeness) and negative (conflict and dependence) aspects of the relationship. Parenting stress and caregiving burden were included as mediator 1 and mediator 2, respectively. Although the sample size may raise concerns about the robustness of the mediation model based on conventional rules of thumb (Kline, 2023, p. 16), recent evidence suggests that such models can perform adequately even with small samples ranging from 50 to 150 participants (Aldred et al., 2012; Schiltz et al., 2021). Therefore, the use of this statistical approach was deemed appropriate.

### 2.2. Participants

This study included a sample of 51 parents of autistic children aged between 4 and 18 years. Table 1 shows the sociodemographic characteristics of the parents and children involved.

### 2.3. Measures

#### 2.3.1. Potential Predictor

*Parental resolution*. The 42-item Reaction to Diagnosis Questionnaire (Sher-Censor et al., 2020) is a parent-report measure assessing parental resolution compared to lack of resolution regarding a child’s diagnosis. The instrument is based on the theoretical framework developed by Marvin and Pianta (1996). For the purpose of this study, the translated Italian version of the questionnaire (Cronbach’s α = 83) was used (Lecciso et al., 2025a). The scale includes 13 items reflecting the resolution of the diagnosis (e.g., “Today I can see my child’s difficulties as well as their strengths and achievements”; “I feel that my feelings regarding my child’s diagnosis have changed since my child received the diagnosis”) and 29 reverse-coded items indicating a lack of resolution (e.g., “I am angry about everything that happened to my child and me”; “It is difficult for me to stop thinking about my child’s diagnosis and difficulties”). Response options ranged from 1 (“strongly disagree”) to 5 (“strongly agree”). A total score was calculated as the average of all items, with higher scores indicating a higher degree of resolution.

#### 2.3.2. Mediators

*Parenting stress*. The 21-item Depression Anxiety Stress Scale (Bottesi et al., 2015) is a self-report questionnaire assessing perceived levels of depression, anxiety, and stress. For the purpose of this study, only the stress subscale was used (e.g., “I feel stressed”; “It is difficult to relax”). Response options ranged from 0 (“never”) to 3 (“almost always”). The stress score was calculated as the average of the relevant items, with higher scores indicating higher perceived stress. The Italian version of the scale (Bottesi et al., 2015) was administered (Cronbach’s α = 0.87).

*Caregiving Burden*. The 22-item Zarit Burden Inventory (Zarit et al., 1980) is a caregiver-report questionnaire assessing perceived burden associated with caring for a family member with a disability. Example items include “Do you feel stressed between caring for your relative and trying to meet other responsibilities for your family or work?” and “Are you afraid of what the future holds for your relative?”. Response options ranged from 0 (“never”) to 4 (“almost always”). A total score was computed by averaging the responses, with higher scores indicating greater caregiving burden. The Italian version of the measure (Chattat et al., 2011) was used (Cronbach’s α = 0.90).

#### 2.3.3. Outcome

*Quality of the Parent*–*Child Relationship*. The 26-item Child–Parent Relationship Scale (Pianta, 1992) is a parent-report questionnaire assessing parents’ perceptions of their relationship with their child. The scale measures both positive and negative aspects of the parent–child relationship through three dimensions: Closeness, Conflict, and Dependence. Closeness is considered a positive aspect of the relationship and is assessed through items such as “If upset, my child will seek comfort from me” and “My child spontaneously shares information about themselves”. Conflict is considered a negative aspect of the relationship and is measured with items such as “My child and I always seem to be struggling with each other” and “Dealing with my child drains my energy”. Dependency is considered a stressful feature of the attachment relationship and is assessed with items such as “My child reacts strongly to separation from me” and “My child is overly dependent on me”. Response options ranged from 1 (“definitely does not apply”) to 5 (“definitely applies”). Three subscale scores were calculated by averaging the items, with higher scores indicating higher levels of closeness (Cronbach’s α = 0.70), conflict (Cronbach’s α = 0.82), and dependency (Cronbach’s α = 0.67). In addition, a total score was calculated by summing all the items. The Italian version of the scale (Rinaldi et al., 2023) was used in this study.

## 3. Results

### 3.1. Preliminary and Descriptive Analyses

The Gaussianity of the data distribution was assessed using the Shapiro–Wilk test (W). The data were normally distributed for the total resolution score (*W* = 0.973; skewness = −0.280; kurtosis = 0.951), caregiving burden (*W* = 0.982; skewness = 0.094; kurtosis = −0.139), and the negative aspects of the parent–child relationship—specifically conflict (*W* = 0.960; skewness = 0.502; kurtosis = 0.865) and dependence (*W* = 0.971; skewness = 0.186; kurtosis = −0.610).

The data were normally distributed for the parenting stress score (*W* = 0.953; skewness = 0.625; kurtosis = 0.122) and the positive aspect of the parent–child relationship—closeness (*W* = 0.937; skewness = −0.698; kurtosis = 0.094).

Levene’s test for equality of variances showed that variances across groups were not homogeneous. Based on preliminary exploratory analyses, nonparametric group comparisons were conducted.

Table 2 shows the descriptive statistics for the study variables.

Descriptive statistics indicated that the levels of stress reported by parents were generally non-clinical. The mean score for parental resolution was in the medium-to-high range. Regarding parenting stress and caregiving burden, mean values fell within the mild to moderate range of caregiving strain (Zarit et al., 1980). As for the quality of the parent–child relationship, parents reported a close, low-conflict, and low-to-moderately dependent relationship with their child. The overall quality of the parent–child relationship did not appear to be clinically concerning. No clinical or borderline scores were observed across the study variables.

### 3.2. Group Comparisons

A Mann–Whitney *U* test was performed to compare parental resolution, parenting stress, caregiving burden, and the positive (closeness) and negative (conflict and dependence) aspects of the parent–child relationship between parents of boys and parents of girls. A significant difference emerged only for the negative aspect of conflict (*U* = 113.5; *p* = 0.030), with parents of boys reporting a more conflictual relationship [*M* (*SD*) = 31.17(7.81)] than parents of girls [*M* (*SD*) = 24.9(7.01)].

In addition, a Mann–Whitney *U* test was conducted to compare the study variables based on parents’ gender and children’s age group (childhood, preadolescence, and adolescence). No significant differences were found for any of the study variables.

### 3.3. Correlational Analysis

Pearson’s correlations were computed to examine the relationships between the study variables and the participants’ sociodemographic characteristics (i.e., parents’ and children’s age). Table 3 reports the results. 

Parental resolution was positively associated with closeness and negatively correlated with conflict in the parent–child relationship.

Parenting stress and caregiving burden were both positively associated with the negative aspects of the parent–child relationship—conflict and dependence. While parenting stress was negatively associated with parental resolution, it was positively correlated with closeness. Similarly, caregiving burden was negatively associated with parental resolution and positively correlated with closeness.

No significant associations were found between the study variables and either the parents’ or children’s age.

### 3.4. Multiple Mediation Model

Due to the lack of significant associations between sociodemographic characteristics and the study variables, the multiple mediation model was tested without covariates. More specifically, correlations showed that neither parents’ nor children’s age were associated with parental resolution, parenting stress, caregiving burden, or any dimension of the parent–child relationship. The comparisons by child’s gender showed a significant difference for only one aspect of the quality of the parent–child relationship—conflict. As the outcome of the multiple mediation model was an aggregate variable consisting of the closeness, conflict, and dependence dimensions, the effect of child’s gender may have been obscured. Based on these findings, the hypothesised multiple mediation model (Figure 1) was tested without covariates.

The multiple mediation model showed a significant total effect (β = 0.012; BootLLCI = 0.002; BootULCI = 0.024) and a significant direct effect of parental resolution on the parent–child relationship (β = 0.223; BootLLCI = 0.058; BootULCI = 0.389). Despite the small effect size, the mediation model suggests that parental resolution of the child’s diagnosis could be a potential protective factor helping parents to perceive the parent–child relationship as close, low in conflict, and characterised by low dependence.

As for the role served by the two mediators—parenting stress (mediator 1) and caregiving burden (mediator 2)—only parenting stress showed a significant, albeit small, indirect effect (β = −0.130; BootLLCI = −0.009; BootULCI = −0.291). This means that the parenting stress may mediate the direct path: the higher the parental resolution, the higher the quality of the parent–child relationship via the lower levels of parenting stress. Regarding the second mediator, the results reported that the caregiving burden did not mediate the direct path. Nevertheless, the path between the parental resolution and the caregiving burden is significant. This means that the parental resolution may be a potential factor decreasing the negative impact of providing care to their autistic child. In sum, the results outlined that the parental resolution may be not only a potential buffer in decreasing the parenting stress levels and the caregiving burden but also a resource in improving the quality of the relationship with their child. Among the mediators, only the parenting stress served a significant role, suggesting further investigations on the role played by the caregiving role.

**Figure 2:**
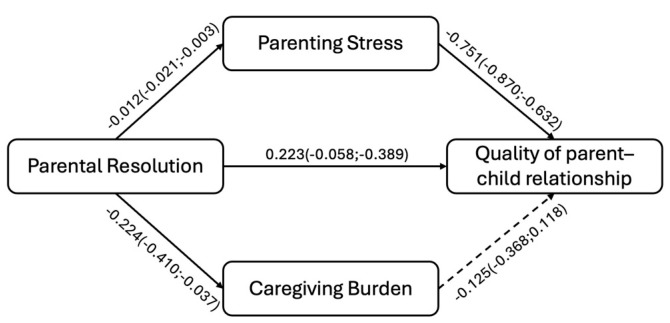
Tested multiple mediation model.

Figure 2 shows the beta coefficients and the corresponding bootstrap confidence intervals.

## 4. Discussion and Future Directions

This study aimed to examine the potential predictive role of parental resolution as a personal resource in fostering a high-quality parent–child relationship in the context of autism. Parenting stress and caregiving burden were tested as mediators in the pathway from parental resolution to the quality of the parent–child relationship, in terms of higher closeness, lower conflict, and reduced dependence. While the impact of parenting stress on the quality of the parent–child relationship and its detrimental effects (Yesilkaya & Magallón-Neri, 2024) have been widely demonstrated, little is known about the role of caregiving burden. In addition, limited research has investigated the potential role of parenting stress and caregiving burden as mediators in the pathway from parental resolution to the quality of the parent–child relationship. Therefore, this study aimed to explore the relationship between parental resolution and the quality of the parent–child relationship by simultaneously examining the roles of two parental risk factors, i.e., parenting stress and caregiving burden.

Based on gender discrepancy in autistic profiles (Napolitano et al., 2022), the preliminary results of this study indicated that parents of male children experienced a more conflictual parent–child relationship than parents of female children. The more pronounced and severe autistic symptoms, along with the higher prevalence of externalising comorbidities (Werling & Geschwind, 2013) observed in males compared to their female counterparts, could be a possible explanation for the result. A potential sequel is the parental perception of the relationship with their child as characterised by discordant and dysfunctional interactions. Nevertheless, it is worth noting that these results contrast with those of other studies (Zamora et al., 2014), which found that parents of autistic girls reported greater dysfunction in parent–child interactions compared to parents of boys. However, this discrepancy may stem from the challenges involved in recognising and managing the subtler autistic symptoms typically exhibited by females. The lack of consistent gender differences in this study may also be attributable to the wide age range and heterogeneity in levels of functioning among participants—two factors that should be considered in future investigations.

### 4.1. The Main Model

With regard to parental perceptions of resolution, stress, caregiving burden, and the quality of the parent–child relationship, the results showed no significant gender differences. These findings are not consistent with previous evidence, which highlighted that mothers report greater warmth, affection, and involvement with children compared to fathers (Fingerman et al., 2020), as well as higher levels of stress (Phetrasuwan & Shandor Miles, 2009) and heavier caregiving burden due to their primary caregiving role (van Niekerk et al., 2023). Nevertheless, the unbalanced distribution of parental gender in the sample led to a cautious interpretation of the results.

The associations identified and the results from the multiple mediation model provided pivotal insights into the research questions, indicating that parental resolution may be a personal resource in fostering a high-quality parent–child relationship. It may also serve as a potential buffer in reducing levels of parenting stress. Parents who have achieved resolution may be more aware of their child’s functioning, which may help them to manage stress more effectively, thereby improving the quality of the parent–child relationship. It is worth noting that resolution of the diagnosis was also negatively associated with caregiving burden. However, caregiving burden did not mediate the pathway between parental resolution and the quality of the parent–child relationship. Therefore, further investigation into the direct and indirect effects of parental risk factors is needed.

Parental attitudes have been shown to play a crucial role in fostering an affective parent–child relationship in both typically developing (Wang, 2023) and autistic children (Seskin et al., 2010). Such relationships, in turn, positively influence autism-specific intervention outcomes (E. Smith et al., 2022). Accordingly, the findings of this study prompt several important reflections.

#### 4.1.1. The Role of Parenting Stress and Caregiving Burden

To begin with, parenting stress is a risk factor that negatively affects the quality of the parent–child relationship. The findings of this study align with evidence: a higher level of parenting stress may distort the parental perception of the relationship, experiencing less warmth, more conflict (Hickey et al., 2020), and particularly a dependent (McStay et al., 2014) relationship. These findings do not seem surprising, given the autistic traits and profiles observed in the sample. Autism-related traits (Pastor-Cerezuela et al., 2016) may affect the parental ability to address everyday challenges, perceiving themselves as incompetent and ineffective in understanding their child’s needs. The latter, whose needs are not met, may become defiant and/or show overt opposition, undermining the quality of the parent–child relationship and increasing parenting stress.

Regarding the role of caregiving burden, the findings of this study showed a strong correlation between caregiving burden and both parental resolution and the quality of the parent–child relationship. The mediation model supported the association between parental resolution and caregiving burden, suggesting that the resolution of the child’s diagnosis could be a potential protective factor in perceiving low stress and/or burden due to the care provided to their autistic child. However, caregiving burden did not impact the parent–child relationship and, thus, it did not significantly mediate the direct path. It is an unexpected result due to the cascade effects the caregiving burden has on parents’ lives, in terms of lower parental quality of life (Marsack-Topolewski & Church, 2019) and reduced life satisfaction (Cetinbakis et al., 2020). Nevertheless, the paucity of studies examining this relationship and the exploratory nature of the current study suggest further investigations.

#### 4.1.2. The Role of Parental Resolution

The challenges experienced by families of autistic children may become particularly significant when parents lack the strategies and resources to cope with autism-related demands (Romero et al., 2021). This may result in burnout (Ren et al., 2024), with parents feeling overwhelmed and exhausted due to their caring for their autistic child and addressing autism-related challenges. To prevent parental burnout with detrimental effects on disengaged parenting behaviours and/or negative parent–child interactions, parental resources should be considered a key aspect of parental mental health. This study highlighted the essential role played by a positive parental attitude following a child’s autism diagnosis—a finding also supported by previous research (Naicker et al., 2023)—with parental resolution being considered a personal resource. The multiple mediation model tested in this study showed that parental resolution directly improved the quality of the parent–child relationship. Therefore, resolution of the diagnosis may serve as a parental resource that can be leveraged in intervention programmes, not only to foster a positive parent–child relationship, but also to improve relationships between parents and siblings (Lecciso et al., 2025a), and between children/adolescents with autism and their siblings (Lecciso et al., 2025a). Parental awareness of their child’s autistic profile may reduce the parents’ stress levels, as it was found in this study. The tested multiple mediation model showed that parents who achieved resolution perceived the relationship with their child as close, non-conflictual, and characterised by low dependence, which supported the protective role of the resource.

According to ecological systems theory (Bronfenbrenner & Morris, 2007), the family is the primary context fostering early developmental processes. Stressed and unresolved parents who lack effective coping strategies to manage caregiving burden may struggle to serve as a secure base for their child. They may also tend to withdraw and avoid seeking professional support. The consequences of such an attitude may extend across personal, social, and family domains. Therefore, promoting the well-being of all family members is both a research priority and a public health imperative, especially when disability is involved.

## 5. Practical and Clinical Implications

The results of this study inform both scholars and clinicians. While scholars should focus their efforts on further investigating the hypothesised model, clinicians are encouraged to develop intervention programmes aimed at mitigating the detrimental effects associated with parents receiving an autism diagnosis for their child.

Regarding the research field, future studies could test the hypothesised mediation model on a larger and more gender-balanced parental sample. Although this study found no significant gender differences between mothers and fathers, prior research has demonstrated discrepancies in maternal and paternal attitudes towards resolution, stress, and caregiving burden (Ozturk et al., 2014). Additionally, future studies could test the hypothesised model on a larger, gender-balanced child sample or gender-specific groups. Due to the gender differences observed in autism profiles (Ferri et al., 2018), examining parental dispositions about the child’s gender may help to develop more targeted intervention programmes.

In terms of clinical implications, the findings of this study align with previous evidence (Wachtel & Carter, 2008), showing that parent training improved positive behavioural interactions between parents and their autistic children, increased satisfaction with parenting and social engagement, and reduced parenting stress and aggression. These results suggest that the parent–child relationship may benefit from positive parental resources, such as resolution of the child’s diagnosis. However, a recent scoping review (Sher-Censor & Shahar-Lahav, 2022) has reported that approximately 70% of parents of autistic children struggle to resolve the diagnosis. Mothers who had accepted the autism diagnosis and achieved resolution demonstrated greater cognitive and emotional involvement during play with their children (Wachtel & Carter, 2008). Furthermore, a high-quality mother–child relationship, characterised by maternal warmth and praise, has been associated with fewer comorbidities and reduced impairments in social reciprocity and repetitive behaviours (L. E. Smith et al., 2010).

**Figure 3:**
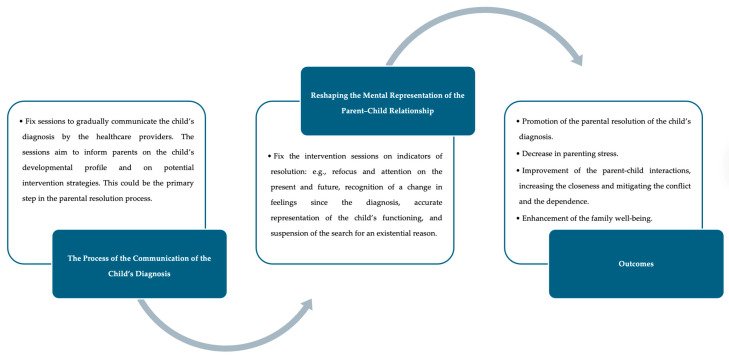
Practical pathways for intervention.

Based on these findings, fostering parental resolution should be a primary aim for professionals throughout the diagnostic process and starting from the diagnosis. During the diagnosis communication process, clinicians can share with parents detailed information regarding the child’s developmental profile (Lecciso et al., 2025c) to increase their awareness about the child’s strengths and challenges. In addition, from the initial stages of shock and denial, professionals can support parents in reshaping their mental representation of the parent–child relationship. Helping parents to integrate their idealised image of the child with the real child may facilitate acceptance of the autism diagnosis. As a result, parents may begin to perceive their child more accurately and realistically, becoming more aware of the child’s functioning in terms of strengths and challenges. Not least, being present during the child’s intervention sessions, as planned by the Parent-delivered Early Start Denver Model (Jhuo & Chu, 2022; Rogers et al., 2012), can support the parental resolution process. Parents not only see the child’s enhancements but also learn strategies to implement early interventions in the home setting (Waddington et al., 2020), promoting the resolution process. Also, parent coaching sessions discussing the challenges faced by parents in everyday life settings can support the resolution process. The professionals can help parents to carefully observe the child’s behaviours, understand the stimulus that triggers them, and provide parents with the strategies to grapple with the complex interplay among the child’s experiences and behaviours, and environmental factors (Siller et al., 2018). Achieving resolution can also enable parents to adopt effective strategies for addressing a large number of everyday challenges, enhancing their sense of parental competence (Levante et al., 2025). Figure 3 summarises practical pathways for clinical intervention.

The timing of diagnosis is critical for promoting resolution (Okoye et al., 2023): the earlier the diagnosis, the greater the opportunity for positive adjustment and effective resolution among family members (De Carlo et al., 2024; Lecciso et al., 2025a; Martis et al., 2024). Efficient screening using reliable measures (Ali et al., 2025; Lecciso et al., 2019), as recommended by guidelines (Subramanyam et al., 2019), should be prioritised by health policymakers as part of preventive care. Early screening can drive positive cascade effects across the entire diagnostic process.

## 6. Limitations and Strengths

Although promising, the results of this study should be interpreted in light of several limitations. Firstly, the small and gender-unbalanced sample of parents and autistic children/adolescents restricts the generalisability of the findings regarding fathers and autistic females. Autism severity was not considered, which limits the understanding of how this variable may affect parental dispositions. The study variables were assessed through parent reports, which reduced data variance. Although recruiting fathers and autistic girls remains a research challenge, future studies with larger and more gender- and age-balanced samples or subsamples are necessary to provide crucial insights to develop effective intervention programmes. Future research could also examine the proposed pathways using different measures and considering the impact of the child’s gender on each dimension of the study variables (e.g., positive and negative aspects of the parent–child relationship)

One of the strengths of this study was its in-depth investigation of the role of parenting stress in shaping the quality of the parent–child relationship, along with the inclusion of two underexplored parental dispositions: caregiving burden and resolution of the child’s diagnosis.

In conclusion, despite the preliminary and exploratory nature of this study, the results pave the way for further research not only on risk factors but also on protective factors influencing vulnerable populations. Both cross-sectional and longitudinal studies should be conducted in order to explore the role of personal resources in coping with challenging conditions. Consequently, intervention programmes should be developed to support healthy family dynamics.

Note: The authors used identity-first (i.e., autistic child) and strengths-based language when referring to the sample.

## Figures and Tables

**Table 1:** Sociodemographic characteristics of the sample.

	Mean/n	SD/%	Range
Parents of Autistic Children			
Gender			
Mothers	44	86.3%	
Fathers	7	13.7%	
Age (years)	43.86	5.97	25–58
Marital status			
With a partner	40	78.6%	
Without a partner	11	21.6%	
Education level			
Low	3	5.9%	
Intermediate	42	82.4%	
High	6	11.7%	
Source of child’s diagnosis communication			
My partner	1	2.2%	
Healthcare providers	30	58.6%	
Myself	18	35.3%	
I asked others	2	3.9%	
**Autistic Children**			
Gender			
Females	10	19.6%	
Males	41	80.4%	
Age (years)	10.96	3.49	4–17
Age range			
Childhood	24	47%	
Preadolescence	13	25.5%	
Adolescence	14	27.5%	
Age at diagnosis (months)	42.71	18.5	18–108
Birth order			
First born	11	21.6%	
Second born	32	62.7%	
Third born or later	8	15.7%	

Note: Low education level = up to 8 years of education; Intermediate education level = up to 13 years of education; High education level = 13 or more years of education.

**Table 2:** Descriptive statistics for the study variables.

Study Variables	*M* (*SD*)	Theoretical Range
**Potential Predictor**		
Resolution Total Score	3.67 (0.39)	1–5
**Mediators**		
Parenting Stress	0.91 (0.54)	0–3
Caregiving Burden	33.68 (14.49)	0–88
**Outcome**		
Quality of Parent–Child Relationship		
Closeness	17.25 (3.87)	1–25
Conflict	29.94 (7.99)	1–70
Dependence	10.27 (3.24)	1–20
Total score	85.04 (11.35)	1–115

**Table 3:** Pearson correlations between study variables and participants’ sociodemographic characteristics.

	(1)	(2)	(3)	(4)	(5)	(6)	(7)	(8)
Parental resolution	−0.349 **	−0.464 ***	0.497 ***	−0.437 ***	−0.169	0.526 ***	−0.017	0.024
Parenting Stress (1)		0.692 ***	−0.471 ***	0.464 ***	0.376 **	−0.594 ***	0.055	0.042
Caregiving burden (2)			−0.383 **	0.441 ***	0.435 ***	−0.565 ***	0.080	0.005
Closeness (3)				−0.286 *	0.042	0.530 ***	−0.259	−0.244
Conflict (4)					0.442 ***	−0.928 ***	−0.116	0.099
Dependence (5)						−0.582 ***	−0.036	−0.197
Quality of the parent–child relationship (6)							0.003	−0.096
Parents’ age (7)								0.322 *
Child’s age (8)								-

* *p* < 0.050; ** *p* < 0.010; *** *p* < 0.001.

## Data Availability

The dataset is available from the authors upon reasonable request.
